# Public sunscreen dispensers and sun-protective behaviours: an observational study in Toronto, Canada

**DOI:** 10.1016/j.pmedr.2026.103485

**Published:** 2026-04-29

**Authors:** Anojini Ravichandran, Jasmin Bhawra

**Affiliations:** School of Occupational and Public Health, Toronto Metropolitan University, 350 Victoria St, Toronto, Ontario M5B 2K3, Canada

**Keywords:** Sun protection, Sun protective behaviours, Sunscreen, Skin cancer, Observational study, Environmental UV, Solar ultraviolet radiation

## Abstract

**Objectives:**

Skin cancer is a growing global health concern, primarily caused by ultraviolet radiation. Although guidelines exist for sunscreen use and other sun-protective behaviours, adherence remains inconsistent. Public sunscreen dispensers increase access, but their association with behavioural change is unclear. This study examined the association between public sunscreen dispensers and observed sun-protective behaviours in Toronto, Canada.

**Methods:**

A cross-sectional, observational study was conducted from July to September 2024 across 25 sunscreen dispensers in Toronto, Canada. A Sun Safety Score (max score = 6) was developed based on six ultraviolet protective behaviours: sunscreen, hats, sunglasses, upper- and lower-body coverage, and closed-toe footwear. Regression models examined the association between dispenser vicinity and sun safety behaviours.

**Results:**

A total of 140 users were observed using dispensers, while 1152 individuals were observed near (<250 m) and far (>250 m) from dispensers. Children and teens showed consistently lower adherence to each sun-protective behaviour than adults. Sun Safety Scores were lower at beaches than parks (2.03 vs. 3.06), and adherence was higher in groups than when using the dispenser alone (2.65 vs. 2.45).

**Conclusions:**

While public sunscreen dispensers increase public access, they are insufficient as a standalone sun safety strategy. Health interventions which combine education and environmental prompts may improve sun-protective behaviours.

## Highlights

• Dispensers improved access but not overall sun-protective behaviour.
• Children and teens showed the lowest levels of sun protection.
• Beaches and hot weather were linked to lower sun protection.
• Groups showed better sun safety behaviours than individuals.
• Behaviour varied by age, location, temperature, and environment.

## 1 Introduction

Skin cancer remains a major global public health concern, with more than 1.5 million non-melanoma and 330,000 melanoma cases reported worldwide in 2022 (Ferlay et al., 2024). Melanoma, the most lethal form, results from the uncontrolled growth of melanocytes, which are the skin cells responsible for producing melanin, primarily due to prolonged exposure to ultraviolet radiation (Boutros et al., 2024).

While skin cancer rates are highest in Australia and Northern Europe (Zhou et al., 2025), countries like Canada, which has a varied climate and ultraviolet levels ranging from 0 in winter to over 10 in summer accounting for more than 60% of global melanoma cases (Brenner et al., 2024; Environment and Climate Change Canada, 2024). In Ontario, an estimated 4750 new melanoma cases were reported in 2024, with higher incidence among men (2800) than women (1950) (Brenner et al., 2024). As the southernmost province, Ontario experiences higher ultraviolet exposure, increasing the risk of skin damage (Statistics Canada, 2023).

Global and national health organizations, including the World Health Organization (WHO) and the Canadian Dermatology Association, recommend sunscreen use, protective clothing, seeking shade, and avoiding peak ultraviolet hours to reduce risk (World Health Organization, 2017; Canadian Dermatology Association, 2025). However, adherence remains inconsistent, particularly in leisure settings like parks and beaches (Ghazawi et al., 2019). Research shows that although 79% of Canadians report using sunscreen occasionally, more than half apply it irregularly, and only 55% avoid peak ultraviolet hours (Canadian Dermatology Association, 2023).

Sunscreen adherence is shaped by factors such as cost, accessibility, forgetfulness, dislike of texture or appearance, and beliefs about susceptibility to skin cancer (Pearlman et al., 2021). In contrast, behaviours like seeking shade or wearing protective clothing are influenced by comfort, weather, and fashion preferences (Ford et al., 2020). However, public sunscreen dispensers have been introduced in several regions, including the United States, Australia, and parts of Europe, to reduce barriers and promote equitable access to sun protection (Wood et al., 2017; Ford et al., 2020; Farag et al., 2025).

The *Be Sun Safe* program is a citywide intervention in Toronto, Ontario (Be Sun Safe, n.d.) that launched 50 free sunscreen dispensers in high-traffic outdoor areas in 2024. Previous research shows that sun safety resources placed near outdoor activity areas, such as pools and worksites, significantly increase sunscreen use (Elliott et al., 2009; Cure et al., 2015). This reflects a behavioural principle consistent with the Health Belief Model (HBM), which suggests that close vicinity to health-promoting resources can prompt use, and dispensers can reduce barriers (i.e., cost, forgetfulness) to sunscreen use (Glanz and Bishop, 2010). While earlier studies have explored dispenser feasibility and sunscreen use (Wood et al., 2017; Ford et al., 2020; Farag et al., 2025), less is known about their broader influence on other sun-protective behaviours. Thus, this study aimed to examine the relationship between the presence and use of public sunscreen dispensers and multiple sun safety practices:

i. Examine how being in close vicinity to public sunscreen dispensers influences sun safety behaviours.
ii. Compare Sun Safety Scores of individuals using public sunscreen dispensers across sociodemographic and environmental factors.
iii. Compare the ground, visibility and accessibility of the sunscreen dispenser to the user count.

## 2 Methods

### 2.1 Study design and setting

**Fig. 1:**
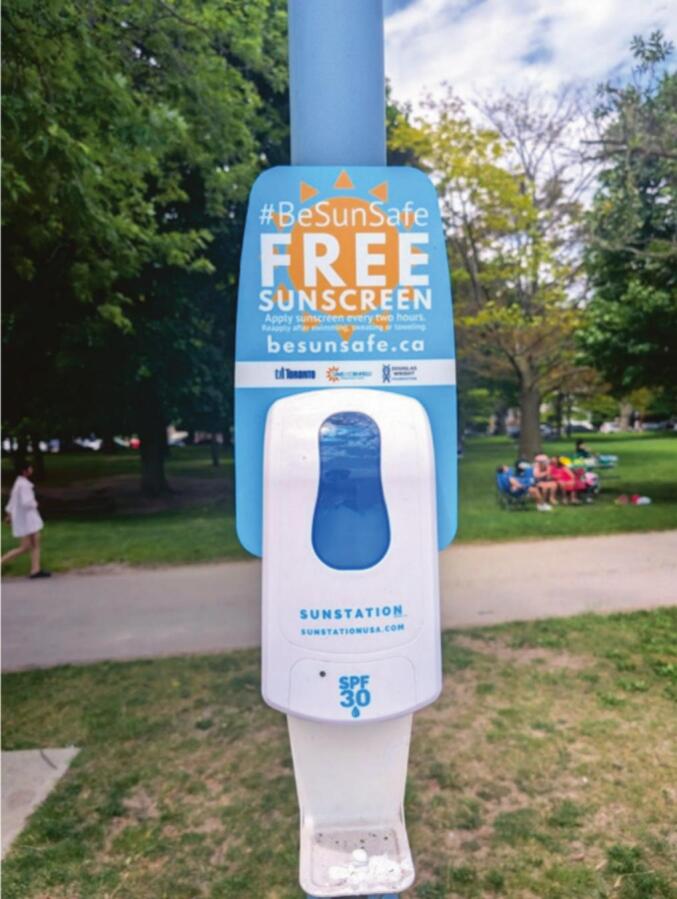
Public sunscreen dispenser used in the Be Sun Safe program in a public park setting in Toronto, Canada, July to September 2024. Image source: Anojini Ravichandran.

A cross-sectional naturalistic observational study was conducted in Toronto, Ontario – the largest city in Canada – with a population of 2.8 million (Statistics Canada, 2023). Toronto has 11 public beaches and over 1500 parks (City of Toronto, n.d). From the 50 sunscreen dispensers installed by the Be Sun Safe program across 5 beaches and 15 parks, a total of 25 were selected via convenience sampling (Be Sun Safe, n.d.) (Fig. 1).

**Fig. 2:**
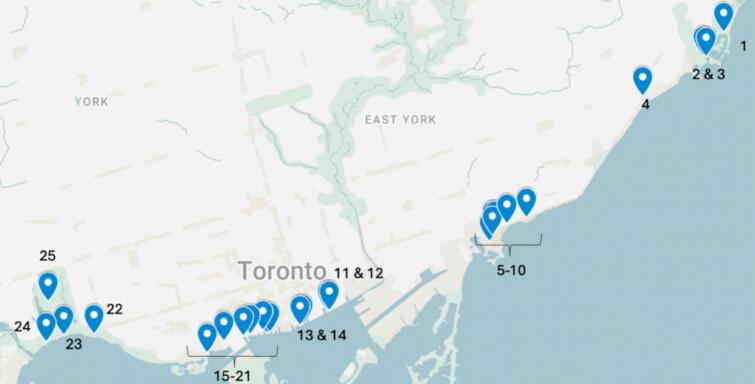
Public sunscreen dispenser used in the Be Sun Safe program in a public park setting in Toronto, Canada, July to September 2024. Image source: Anojini Ravichandran.

Dispensers contained SPF 30, broad-spectrum, mineral sunscreen with 21% zinc oxide (Be Sun Safe, n.d.). They were checked by the observer to confirm that they were stocked and functioning before conducting the observational study, and if not, another dispenser was chosen before making the final shortlist (see Fig. 2). Observations took place on weekdays and weekends from July 12 to August 30, 2024, during two periods per day (morning: 9:00–11:59 AM and afternoon: 12:00–4:00 PM), aligning with peak ultraviolet hours (Dixon et al., 2008). The observer recorded data for one hour per period at each dispenser.

### 2.2 Measures

Data collection instruments were adapted from validated observational studies on sun safety behaviours (Haynes et al., 2022; Rydz et al., 2021). The Sunscreen Dispenser Observation Tool (SDOT) recorded dispenser users' estimated age, gender, hat type, clothing, sunglasses use, sunscreen application by body region, and footwear. The Vicinity-Based Sun Behaviour Observation Tool (VSBOT) assessed general sun-protective behaviours in two zones near the dispensers using a tally-based format (see Appendix I and J). This allowed the observer to efficiently record the behaviours of multiple individuals in a short period.

To evaluate the impact of sunscreen dispenser presence, control zones were defined as areas more than 250 m away from a dispenser with similar activity levels but no dispenser, while dispenser zones were within 250 m of a dispenser. This helped isolate the effect of the dispensers from other factors. The VSBOT instrument was applied in both zones and used at 10 dispenser locations with sufficient space to observe both near and far zones. Observations were conducted during a single time window (12:00 p.m. to 4:00 p.m.) to ensure consistency and align with peak ultraviolet exposure hours (Dixon et al., 2018). This approach is consistent with previous studies examining how the vicinity influences health behaviours (Kaczynski et al., 2008; Troped et al., 2010).

Weather data, including cloud coverage, temperature, and UV index, were collected from Environment and Climate Change Canada (Environment and Climate Change Canada, 2024). Both instruments also recorded dispenser characteristics, including accessibility, visibility, location type and ground type.

Both instruments were reviewed by the research team and piloted before use (see Appendix I and J). Revisions improved category clarity and standardized vicinity measures. The pilot included one hour per time period, with 6 dispenser users and 111 individuals observed across the vicinity zones.

The study protocol was reviewed by the Toronto Metropolitan University Research Ethics Board (REB 2024–129) and was deemed exempt from requiring ethics approval.

VSBOT focused on sun protective behaviours observed from individuals across the vicinity zones **(**Appendix I), recorded as tally counts. Behaviours included wearing hats, sunglasses, long sleeves, long pants, closed-toe shoes, personal sunscreen use, and seeking natural or portable shade.

SDOT focused on individual sunscreen dispenser users and recorded categorical variables including age group (child, teen, adult), gender (man or woman), and hat type (no hat, visor, cap, or broad-brimmed). Additional details included sleeve length (sleeveless to ¼ length, ½ length, ¾ length or longer), leg coverage (bikini/shorts, mid-thigh/knee, or full cover), and sunglasses use (yes or no). Sunscreen application was recorded by body region (arms, face, chest, back, thighs, and lower legs), and footwear as closed-toed (yes or no).

A composite Sun Safety Score was created to quantify protective behaviours among dispenser users for SDOT. It combined six behaviours: sunglasses, hat type, upper-body coverage (at least half-sleeves), lower-body coverage (at least knee-length), visible sunscreen (0.2 points per region, up to 1 point), and closed-toe shoes. Each non-sunscreen behaviour contributed 1 point, for a total score from 0 to 6. Higher scores reflected greater sun protection.

Single behaviours may not reflect overall sun safety habits; however, research indicates that combining multiple actions into a score helps detect broader patterns (Rydz et al., 2021). In this study, the score was calculated for dispenser users to assess whether using the dispenser was linked to other sun-protective behaviours. This aligns with Be Sun Safe's mission of reducing barriers to sunscreen use, thereby encouraging regular sun protection among the public (Be Sun Safe, n.d.). Consistent with prior observational research, the study applied composite measures to assess patterns of sun-protective behaviours among individuals engaging with the intervention (Walkosz et al., 2008).

Age group (child, teen, adult), gender (man or woman), and group type (individual or group) were estimated by the observer and recorded as categorical variables.

Based on information from Environment and Climate Change Canada, temperature (°C) and ultraviolet index were recorded as numerical variables. Cloud coverage was categorized as 0%, 50%, 75%, or 100%. Additional categorical variables included dispenser characteristics such as accessibility (yes/no), visibility (yes/no), ground type (paved/grass), and location type (beach/park).

### 2.3 Statistical analysis

Descriptive summaries included proportions, means, medians and standard deviations to summarize users' demographics, sun safety behaviours, environmental conditions and dispenser characteristics. Fisher's Exact Test assessed differences in each sun protection behaviour by sociodemographic and weather variables (see Appendix L).

Two sets of regression analyses were conducted. The first set used negative binomial regression to examine the effect of dispenser vicinity on individual sun-protective behaviours. Separate models were run for each behaviour, adjusting for age group, cloud coverage, location type, weekend, and temperature.

Second, to examine the effect of dispenser characteristics, analysis was conducted at the dispenser level. Average user counts were calculated for each dispenser across all observation sessions. Descriptive statistics summarized these averages by ground type, visibility, accessibility, and location type.

Negative binomial model results were reported as incident rate ratios (IRR) with 95% confidence intervals (CI), with significance set at *p* < 0.05. Data was analyzed using R version 4.0.2.

## 3 Results

### 3.1 Effect of vicinity to sunscreen dispensers

In total, 1152 individuals were observed across all sites (Table 1). Among those near dispensers, 299 were adults, 83 children, and 109 teens; in the far zones, there were 360 adults, 92 children, and 117 teens (Table 1).

**Table 1:** Distribution of sun-protective behaviours by age group among individuals observed in public outdoor settings in Toronto, Canada, July to September 2024.

Age Group		Sun safety behaviour *N* (%)					
	Total	Sunglasses	Hats	Long Shirts	Long Pants	Portable Shade	Natural Shade
All	1060	343 (32.4)	407 (38.4)	212 (20.0)	359 (33.9)	221 (20.8)	540 (50.9)
Adult	659	298 (45.2)	314 (47.6)	144 (21.9)	256 (38.8)	161 (24.4)	329 (49.9)
Children	175	8 (4.60)	58 (33.1)	23 (13.1)	31 (17.7)	30 (17.1)	110 (62.9)
Teen	226	37 (16.4)	35 (15.5)	45 (19.9)	30 (13.3)	30 (13.3)	101 (44.7)

Negative binomial regression showed no significant effect of dispenser vicinity on sun-protective behaviours (Table 2). Demographic factors were the strongest predictors, with teens and children engaging in fewer protective behaviours than adults.

**Table 2:** Adjusted incidence rate ratios and 95% confidence intervals for sun-protective behaviours by vicinity to sunscreen dispensers, age group, and environmental factors among individuals observed in public outdoor settings in Toronto, July to September 2024 (Total sample size = 1152).

Variables	Sun safety behaviours					
	Sunglasses	Hats	Long Shirt	Long Pants	Portable Shade	Natural Shade
Vicinity						
Close	0.86 (0.61, 1.21)	0.93 (0.65, 1.33)	0.91 (0.56, 1.50)	0.91 (0.64, 1.28)	0.84 (0.36, 1.98)	0.95 (0.63, 1.43)
Far (reference)	–	–	–	–	–	**–**
Age Group						
Children	0.03 (0.01, 0.06)	0.17 (0.12, 0.26)	0.14 (0.08, 0.27)	0.12 (0.07, 0.19)	0.16 (0.06, 0.43)	0.30 (0.19, 0.48)
Teen	0.13 (0.08, 0.19)	0.11 (0.07, 0.17)	0.27 (0.16, 0.48)	0.27 (0.18, 0.40)	0.20 (0.07, 0.53)	0.28 (0.17, 0.45)
Adult (reference)	–	–	–	–	–	–
Environmental						
UV Index	1.15 (1.01, 1.31)	1.20 (1.06, 1.37)	1.18 (0.98, 1.41)	0.93 (0.82, 1.06)	1.55 (1.08, 2.22)	1.16 (1.00, 1.36)
Cloud Coverage	0.99 (0.98, 1.00)	0.99 (0.98, 1.00)	0.99 (0.98, 1.01)	1.00 (0.99, 1.00)	1.01 (0.99, 1.03)	1.00 (0.99, 1.01)
Weekday	1.03 (0.64, 1.67)	0.66 (0.40, 1.07)	0.71 (0.36, 1.41)	0.97 (0.60, 1.58)	1.92 (0.52, 7.05)	0.49 (0.27, 0.86)
Weekend (reference)	–	–	–	–	–	**–**

Adjusted incidence rate ratios and 95% confidence intervals were estimated using multivariable negative binomial regression models, adjusting for age group, UV index, cloud coverage, and day of observation. *Close* areas were located within 250 m of a sunscreen dispenser, while *Far* areas were located more than 250 m away. Reference groups: *Far* (vicinity), *Adult* (age group), and *Weekend* (day).

### 3.2 Sun-protective Behaviours among sunscreen dispenser users

Among the 25 dispensers, 12 were observed on weekdays, and 13 on weekends. An adult accompanied all the children who used the dispensers. Of 140 users, most were women (71%) and adults (54%); 8 were children (5.7%) (Table 3).

**Table 3:** Proportions of sun-protective behaviours by vicinity to sunscreen dispensers and age group among children, teens, and adults observed in public outdoor settings in Toronto, July to September 2024.

Vicinity	Age Group	Total People	Sunglasses	Hats	Long Shirts	Long Pants	Portable Shade	Natural Shade
Close	All	491	158 (32.18)	179 (36.46)	96 (19.55)	175 (35.64)	99 (20.16)	274 (55.80)
	Adult	299	137 (45.82)	136 (45.48)	69 (23.08)	136 (45.48)	75 (25.08)	173 (57.86)
	Children	83	5 (6.02)	29 (34.94)	15 (18.07)	15 (18.07)	9 (10.84)	59 (71.08)

Far	Teen	109	16 (14.68)	14 (12.84)	12 (11.01)	24 (22.02)	15 (13.76)	42 (38.53)
	All	569	185 (32.52)	228 (40.07)	116 (20.39)	184 (32.34)	122 (21.44)	266 (46.75)
	Adult	360	161 (44.72)	178 (49.44)	75 (20.83)	120 (33.33)	86 (23.89)	156 (43.33)
	Children	92	3 (3.26)	29 (31.52)	8 (8.70)	16 (17.39)	21 (22.83)	51 (55.43)

By age, the mean scores were higher for adults (2.99) than for children (2.60) and teens (2.73). By sex, men had a higher mean score (3.24) than women (2.79) (Fig. 3a). By environmental factors, scores decreased with increasing temperature and ultraviolet index.

### 3.3 Effect of sunscreen dispenser's characteristics

Usage was higher on paved surfaces than on grass (Table 4) and greater at beaches (mean = 2.15, SD = 0.96) than in parks (mean = 1.18, SD = 0.88). Dispensers that were not accessible (mean = 2.39, SD = 1.40) or not visible (mean = 1.93, SD = 1.07) had higher average use.

**Table 4:** Proportions of sunscreen dispenser users by dispenser characteristics among individuals observed in public outdoor settings in Toronto, Ontario, Canada, July to September 2024 (total sample size = 140).

Characteristic	n (%)
	Total Sunscreen Dispenser Users = 140
Ground Type	
Grass	25 (17.86)
Paved	115 (82.14)
Visibility	
Visible	76 (54.29)
Not Visible	64 (45.71)
Accessibility	
Accessible	124 (88.57)
Not Accessible	16 (11.43)
Location Type	
Beach	83 (59.29)
Park	57 (40.71)

n represents the number of sunscreen dispenser users in each category.

% = Proportion of sunscreen dispenser users.

### 3.4 Data exclusion

Four of the 25 dispensers had no users and were excluded from the Sun Safety Score analysis, which included only dispenser users. One dispenser was empty and was replaced with an alternative.

## 4 Discussion

This study assessed the effect of public sunscreen dispensers on sun-protective behaviours in a large Canadian city. While proximity to dispensers did not significantly influence protective behaviours, analyses highlighted important age differences.

Teens exhibited inconsistent behaviour, reflecting a significant gap in sun safety adherence. Existing research suggests that several factors may be responsible for this difference. For example, social norms may disproportionately influence teens, as using sun protection is discouraged through peer pressure and cultural trends that associate tanning with beauty (Patel et al., 2019). Adolescents also perceive themselves as having low invulnerability, underestimating their own risk of skin damage (Gamage et al., 2021; Cokkinides et al., 2001). Cokkinides et al. (2001) found that American adolescents who acknowledged the risk of sun exposure still engaged in intentional tanning, reflecting a gap between perceived susceptibility and behaviour. Additionally, research has shown that many teenagers lack sufficient knowledge about sun safety, including the long-term adverse effects of ultraviolet exposure and effective protective measures (Gamage et al., 2021; Patel et al., 2019). A study found that among 2097 Australian students (aged 12–13), only 39% identified UV radiation as a cause of skin cancer, and just 36% reported regular sunscreen use using a self-administered questionnaire (Gamage et al., 2021). Overall, adolescents engage in lower levels of sun-protective behaviour than adults, a pattern consistently observed across international settings, with a recent literature review synthesizing studies from North America, Europe, and Australia reporting similar trends among youth (Raymond-Lezman & Riskin, 2023). According to HBM, individuals are more likely to adopt protective behaviours when they perceive themselves as susceptible to skin cancer, view the consequences as serious, recognize the benefits of sun safety, and face few barriers to action (Glanz and Bishop, 2010). Teens may view tanning benefits as outweighing the risks of ultraviolet damage and feel less susceptible to long-term effects like cancer (Haynes et al., 2022).

Children demonstrated the lowest sun protection. Although important, sunscreen is frequently used as a stand-alone measure, with parents overlooking hats or shade (Autier et al., 2007). Studies also propose that children may resist certain protective behaviours or not understand their importance (Thoonen et al., 2021). In this study, the inconsistent adherence of children may reflect a low perceived susceptibility to skin cancer and a sense that the consequences are not severe or immediate (Thoonen et al., 2021). Moreover, perceived barriers, such as discomfort from wearing protective clothing or the inconvenience of seeking shade, may further discourage behaviours (Pearlman et al., 2021).

In this study, Sun Safety Scores showed that men engaged in more sun-protective behaviours yet were observed using the dispensers less frequently than women. This contradiction may reflect differing motivations and perceptions around sun safety. Research shows that women place importance on social and health outcomes, such as maintaining skin health and preventing sun damage, often linked to cultural standards of attractiveness (Falk and Anderson, 2013). In a Swedish study of 415 primary care patients, women reported greater readiness to adopt sun protection, due to stronger awareness of ultraviolet risks compared to men (Falk and Anderson, 2013). The HBM further supports this finding, indicating that women may have higher perceived susceptibility and severity regarding skin damage, while men may show lower motivation due to reduced perceived relevance of appearance-related outcomes (Glanz and Bishop, 2010).

Moreover, individuals in groups had higher Sun Safety Scores than those observed alone in this study. According to the HBM, groups enhance cues to action by creating an environment where protective behaviours are encouraged (Raymond-Lezman & Riskin, 2023). Individuals encourage others to wear sunscreen, seek shade, and wear protective clothes when together, increasing perceived benefits and self-efficacy (Tsai et al., 2016). In their systematic review of children's and parents' sun safety attitudes and behaviours, Raymond-Lezman and Riskin (2023) found that social support plays a key role in motivating sun protection, particularly in outdoor group settings where social norms often encourage protective practices. For example, they found that on beaches, friends are more likely to practice sun protection as a group, urging each other to seek shade or wear sunscreen. People who are alone may be less likely to take precautions when no one is present to reinforce the importance of sun safety.

Environmental factors also influenced sun-protective behaviours among dispenser users. Sun Safety Scores were higher on weekends, possibly reflecting fewer time constraints and reduced perceived barriers, consistent with O'Riordan et al. (2008) who, in a study of study in Hawaii with lifeguards, children and parents (*n* = 27), which found that people were more diligent about using sunscreen and seeking shade on weekends. Weekends allow people to focus more on sun protection since they have more time for outdoor activities and less work-related stress. Drawing from HBM, weekends may heighten perceived susceptibility to ultraviolet exposure and provide cues to action, as individuals spend more time outside and encounter more reminders to practice protection (Glanz and Bishop, 2010). In contrast, individuals at beaches had lower scores, possibly due to practical and social barriers like limited clothing and the appeal of tanning (Shuk et al., 2012). The HBM suggests that the inconvenience of wearing protective clothing and the use of only sunscreen becomes more noticeable at beaches (Robinson and Rademaker, 1998). For example, Shuk et al. (2012) interviewed melanoma survivors and discovered reduced sun protection atthe beach, due to the emphasis of tanning and discomfort from physical activity. Similarly, higher temperatures were linked to reduced sun protection, supporting previous findings that discomfort is a major barrier to behaviours like wearing long sleeves (Haynes et al., 2021). Ultraviolet Index (UVI) levels during the study ranged from 2 to 9, corresponding to WHO exposure categories from low (<2) to very high (8–10) (World Health Organization, 2002). Although protection is recommended above UVI 3 and reinforced above 8, Sun Safety Scores did not consistently increase at higher levels. Elevated temperatures on high-UVI days may increase discomfort, amplifying perceived barriers and offsetting perceived susceptibility. This suggests that objective environmental risk alone may be insufficient to prompt behavioural adaptation. Cloud coverage was also examined; however, scores did not significantly vary across conditions. While overcast days were cooler, UVI did not decline proportionally. If individuals rely on visible brightness or ambient heat as cue for risk, cloudy conditions may reduce perceived susceptibility despite high ultraviolet exposure. Within the HBM, behaviour is unlikely to shift if perceived susceptibility remains low and perceived barriers, such as discomfort, remain salient (Glanz and Bishop, 2010).

Sunscreen use varied by dispenser characteristics, with higher use at locations where dispensers were less visible or accessible. Similarly, a Dutch study of outdoor construction workers (*n* = 67) found that dispensers were underused even when visible, suggesting that visibility alone does not drive use (Keurentjes et al., 2022). The HBM suggests stronger perceived risk or more salient cues to action may be needed to encourage consistent use (Glanz and Bishop, 2010).

Findings from this study have broader implications for strengthening this program. Incorporating prompts such as temperature-sensitive signage into programming may encourage protective behaviours among the public during high-risk periods (Walkosz et al., 2008). Secondly, dispenser visibility for sunscreen dispenser programs appears to be insufficient as a standalone intervention; thus, combining features like QR codes, clear instructions, or visual prompts may help translate access into actual use (Farag et al., 2025). Thirdly, the inconsistencies among youth highlight the possible need for targeted campaigns highlighting short-term benefits and appearance-related outcomes. The role of group dynamics presents an opportunity to utilize sun safety ambassadors, families or social media campaigns to promote sun safety (Raymond-Lezman & Riskin, 2023). Setting-specific strategies are also needed. At beaches, interventions could include dispensers alongside free shade structures and informational kiosks, while in parks and other community settings, strategies can include signage and educational efforts.

Findings should be interpreted with caution,. Pre-applied sunscreen and use of personal sunscreen could not be assessed in this observational design; therefore, some individuals categorized as non-users may have already applied sunscreen. Data was collected by a single observer, introducing potential subjectivity. Convenience sampling may limit generalizability, and age and sex estimates based on appearance may have led to misclassification. References to gender-related differences may also not reflect participants' gender identities.

## 5 Conclusions

This study examined how public sunscreen dispensers influence sun-protective behaviours in Toronto, Canada. Demographic and environmental factors were key predictors, with children and teens showing the lowest protection. Findings highlight the need for approaches that combine sunscreen access with education and community engagement. Future studies should assess the long-term impact of integrated strategies that align environmental design with behavioural messaging to reduce UV exposure and skin cancer risk.

## CRediT authorship contribution statement

**Anojini Ravichandran:** Writing – review & editing, Writing – original draft, Visualization, Validation, Software, Resources, Project administration, Methodology, Investigation, Formal analysis, Data curation. **Jasmin Bhawra:** Writing – review & editing, Writing – original draft, Visualization, Validation, Supervision.

## Ethical compliance

The Toronto Metropolitan University Research Ethics Board determined that this study was exempt from formal ethics review (REB 2024-129) based on the nature of the data and study design.

## Funding

The authors declare that no specific grant from funding agencies in the public, commercial, or not-for-profit sectors.

## Declaration of competing interest

The authors declare that they have no known competing financial interests or personal relationships that could have appeared to influence the work reported in this paper.

## Data Availability

Data will be made available on request.
