# Supplementary material for: Public sunscreen dispensers and sun-protective behaviours: an observational study in Toronto, Canada
Source: Prev Med Rep. 2026 Apr 29;66:103485. doi: 10.1016/j.pmedr.2026.103485 (PMC13158563; doi:10.1016/j.pmedr.2026.103485)
Supplement: Supplementary material [file mmc1.docx]

**Appendix**

**Appendix A**

**
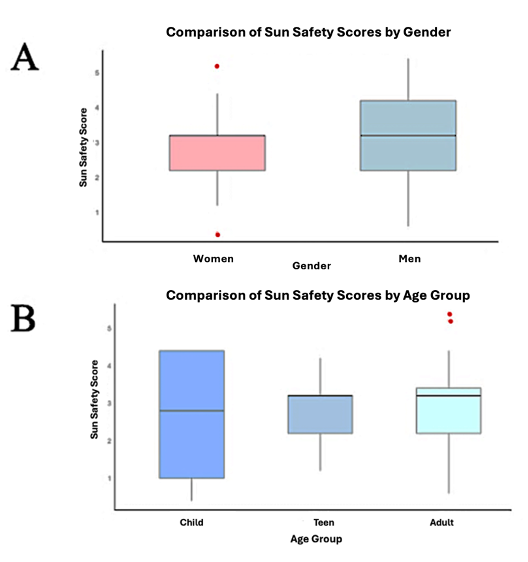
**

**Figure 3.** Distribution of Sun Safety Scores by gender and age group among children, teens, and adults observed in public outdoor settings in Toronto, July to September 2024. The central line in each box represents the median Sun Safety Score, and the box indicates the interquartile range. Whiskers extend to 1.5 times the interquartile range, and outliers are shown as individual points. (A) Distribution of Sun Safety Scores by gender among men and women. (B) Distribution of Sun Safety Scores by age group among children, teens, and adults.

**Appendix B**

**Table 5.** Distribution of sunscreen dispenser users’ characteristics by time of day among adults, teens, and children observed in public outdoor settings in Toronto, July to September 2024.

| Variable |  |  |  |
| --- | --- | --- | --- |
|  | | Morning^a^ | Afternoon^b^ |
|  | (n= 39) | (n= 101) |  |
| Age Group |  |  |  |
| Adults | 30 (76.92) | 76 (75.25) |  |
| Teens | 9 (23.08) | 17 (16.83) |  |
| Children | 0 (0.00) | 8 (7.92) |  |
| Gender |  |  |  |
| Men | 9 (23.08) | 31 (30.69) |  |
| Women | 30 (76.92) | 70 (69.31) |  |

^a^Morning observations were conducted between 9:00–11:59 AM; ^b^Afternoon observations were conducted between 12:00–4:00 PM.

n represents the number of sunscreen dispenser users observed within each time-of-day group.

**Appendix C**

**Table 6.** Proportion of sun-protective clothing and sunscreen application behaviours by time of day among sunscreen dispenser users observed in public outdoor settings in Toronto, July to September 2024.

| % | | |
| --- | --- | --- |
|  | Morning^a^ | Afternoon^b^ |
| Sun Protective Behaviours | (n= 39) | (n=101) |
| Sunglasses | 23.08 | 32.67 |
| Visor | 0.00 | 0.00 |
| Cap | 33.33 | 31.68 |
| Broad-Brimmed Hat | 0.00 | 0.99 |
| Sleeveless Shirt to ¼ length | 17.95 | 5.94 |
| Half-Length Shirt | 41.03 | 51.49 |
| Three-Quarter to Full Length Shirt | 41.03 | 32.67 |
| Bikini/Shorts | 58.97 | 50.50 |
| Mid-Thigh/Knee-Length Pants | 2.56 | 8.91 |
| Full Cover Pants | 38.46 | 40.59 |
| Sunscreen on Arms | 100.00 | 97.03 |
| Sunscreen on Face | 10.26 | 16.83 |
| Sunscreen on Chest | 0.00 | 0.00 |
| Sunscreen on Back | 0.00 | 0.99 |
| Sunscreen on Thighs/Lower Legs | 12.82 | 7.92 |
| Closed-Toed Shoes | 69.23 | 75.25 |

^a^Morning observations were conducted between 9:00–11:59 AM;

^b^Afternoon observations were conducted between 12:00–4:00 PM.

n represents the number of sunscreen dispenser users observed within each

time-of-day group.

%= Proportion of sunscreen dispenser users.

**Appendix D**

**Table 7.** Sun Safety Score descriptive summary across demographic and environmental factors.

| Category | n | Mean Score | M | SD |
| --- | --- | --- | --- | --- |
| Age Group |  |  |  |  |
| Adult | 106 | 2.99 | 3.20 | 0.97 |
| Child | 8 | 2.60 | 2.80 | 1.78 |
| Teen | 26 | 2.73 | 3.20 | 0.89 |
| Gender |  |  |  |  |
| Women | 100 | 2.79 | 3.20 | 0.96 |
| Men | 40 | 3.24 | 3.20 | 1.08 |
| Group Status |  |  |  |  |
| Group | 118 | 2.99 | 3.20 | 1.01 |
| Individual | 22 | 2.54 | 2.20 | 0.95 |
| UV Index |  |  |  |  |
| 2 | 15 | 3.40 | 3.20 | 0.68 |
| 3 | 17 | 2.52 | 2.20 | 0.97 |
| 4 | 28 | 2.93 | 3.20 | 0.94 |
| 5 | 12 | 3.30 | 3.20 | 0.64 |
| 6 | 22 | 3.25 | 3.40 | 1.16 |
| 7 | 26 | 2.92 | 2.30 | 0.90 |
| 8 | 13 | 2.14 | 2.20 | 1.06 |
| 9 | 7 | 2.54 | 2.40 | 1.42 |
| Temperature (°C) |  |  |  |  |
| 21 | 2 | 3.70 | 3.70 | 0.71 |
| 22 | 3 | 2.87 | 3.20 | 0.58 |
| 23 | 4 | 3.25 | 3.20 | 1.32 |
| 24 | 22 | 3.05 | 3.20 | 1.04 |
| 25 | 37 | 3.09 | 3.20 | 0.80 |
| 26 | 18 | 3.21 | 3.20 | 1.17 |
| 27 | 38 | 2.46 | 3.20 | 1.01 |
| 28 | 16 | 2.93 | 3.20 | 1.08 |
| Cloud Coverage (%) |  |  |  |  |
| 0 | 15 | 3.00 | 2.40 | 1.01 |
| 25 | 63 | 2.92 | 3.20 | 0.98 |
| 50 | 62 | 2.90 | 3.20 | 1.06 |
| Time Period |  |  |  |  |
| Morning | 39 | 2.73 | 2.40 | 0.99 |
| Afternoon | 101 | 2.99 | 3.20 | 1.02 |
| Type of Day |  |  |  |  |
| Weekend | 78 | 3.05 | 3.20 | 0.97 |
| Weekday | 62 | 2.75 | 2.40 | 1.05 |
| Location Type |  |  |  |  |
| Beach | 83 | 2.70 | 2.40 | 0.84 |
| Park | 57 | 3.24 | 3.20 | 1.07 |

n represents the number of sunscreen dispenser users in each category. M= median of the central value of the sun safety score distribution. SD = standard deviation.

**Appendix E**

**Table 8.** Dispenser-level usage of sunscreen dispensers by dispenser characteristics among sites observed in Toronto, Ontario, Canada, July to September 2024.

| Characteristic |  |  |  |
| --- | --- | --- | --- |
|  | Sunscreen Dispenser Users (n= 140)^a^ | |  |
|  | Mean User Per Dispenser | SD | Number of Dispensers (N = 25) |
| Ground Type |  |  |  |
| Paved | 1.75 | 1.04 | 20 |
| Grass | 1.04 | 0.75 | 5 |
| Accessibility |  |  |  |
| Yes | 1.50 | 0.95 | 22 |
| No | 2.39 | 1.40 | 3 |
| Visibility |  |  |  |
| Visible | 1.35 | 0.94 | 14 |
| Not Visible | 1.93 | 1.07 | 11 |
| Location Type |  |  |  |
| Beach | 2.15 | 0.96 | 11 |
| Park | 1.18 | 0.88 | 14 |

^a^ The total number of dispenser users observed across all sites.

SD= Standard Deviation

Mean users per dispenser and its standard deviation were calculated across all 25 dispensers, stratified by dispenser site characteristics

N = 25 represents the total number of sunscreen dispensers observed across all sites.

**Appendix F**

**Table 9.** Data dictionary for variables measuring sun-protective behaviours and sunscreen dispenser use among individuals observed using Observation Tool 1 in public outdoor settings in Toronto, July to September 2024.

| Data Point | Description | Type |
| --- | --- | --- |
| Zone locations | Location of the dispenser that is being measured (Close Vicinity/ Far Vicinity) based on whether 250m close to dispenser or farther. | Character |
| Date | Date in which dispenser was observed | Numerical |
| Time Interval | Time dispenser was observed | Numerical |
| Visibility of dispenser | Whether the nearest dispenser is clearly visible, partially or obscured | Character |
| Distance from dispenser (m) | The distance of the nearest dispenser | Numerical |
| Demographic Group | Estimated age group of the observed individuals | Character |
| Count Wearing Sunglasses | Number of people wearing sunglasses | Numerical |
| Count Wearing Hats | Number of people wearing hats | Numerical |
| Count observed applying Sunscreen | Number of people seen applying their own sunscreen | Numerical |
| Count Wearing Long Shirt | Number of people wearing long shirts | Numerical |
| Count Wearing Long Pants | Number of people wearing long pants | Numerical |
| Count using portable shade/ use of shade (Ex: Umbrella) | Number of people using their own portable shade including umbrellas and tents | Numerical |
| Count using natural shade (Ex: trees) | Number of people using trees as shade | Numerical |
| Estimated total people | Total number of people in the location | Numerical |

**Appendix H**

**Table 10.** Data dictionary for additional variables collected using Observation Tool 2 among individuals observed in public outdoor settings in Toronto, July to September 2024.

| Data Point | Description | Type |
| --- | --- | --- |
| User Count | Count of individuals observed during each entry | Numerical |
| Group ID | Identification number for each individual in groups that observed | Character |
| Location | Location name of the dispenser | Character |
| Date | Date in which dispenser was observed | Numerical |
| Time Interval | Time dispenser was observed | Numerical |
| UV index | The UV index at the time of observation | Numerical |
| Temperature | The temperature at the time of observation | Numerical |
| Cloud Coverage | The cloud coverage at the time of observation | Character |
| Demographic group | Estimated age group of the observed individuals | Character |
| Gender | Gender of the observed individuals | Character |
| Sunglasses | Use of sunglasses as a sun protection measure | Character |
| Hat - No Hat | Observation of hat usage | Character |
| Hat - Visor | Specific type of hat observed | Character |
| Hat - Cap | Another specific type of hat observed | Character |
| Hat - Broad Brim | Broad-brimmed hats observed | Character |
| Shirt - Sleeveless to ¼ length | Type of shirt worn of sleeveless to ¼ sleeves | Character |
| ½ Length | Observation of ½ length sleeved shirts | Character |
| Shirt - ¾ Length to full | Observation of ¾ length to full sleeved shirts | Character |
| Leg - Bikini/Shorts | Leg cover type indicating sun exposure | Character |
| Leg - Mid-thigh/Knee | Mid-thigh to knee coverage observed | Character |
| Leg - Full Cover | Full leg coverage observed | Character |
| Sunscreen on Arms | Sunscreen application on upper extremities | Character |
| Sunscreen - Face | Sunscreen application on face observed | Character |
| Sunscreen on Chest | Sunscreen application on chest | Character |
| Sunscreen on Back | Sunscreen application on back | Character |
| Sunscreen - Legs | Sunscreen application on lower extremities | Character |
| Shoes - Closed-Toe | Observation of closed-toe shoes | Character |
| Visibility | The visibility of the dispenser | Character |
| Accessibility | Whether the dispenser is accessible to access | Character |
| Ground Type | The type of ground the dispenser is mounted based paved (concrete or asphalt) or grass (turf or natural grass areas) | Character |
| Location Type | The type of location the dispenser is mounted. Location was classified as 'beach' if the area provided allows direct access to water for recreational use, and as 'park' if not and was primarily used for land-based activities. | Character |
| Weekday | Dispenser observation assessed on weekday | Character |
| Weekend | Dispenser observation assessed on weekend | Character |

**Appendix I**

**Comparative Vicinity Observational Tool for Sun Safety Practices (Observation tool 1)**

**Zone Type:** [ ] Sunscreen Dispenser Zone [ ] Control Zone

**Location of Observation:** _________________________________________________

**Date:** _______________ **Time:** _______________

**Weather and Environmental Conditions:**

**UV Index:** ___________

**Temperature:** ________°C

**Cloud Coverage:**

- 0%
- 25%
- 50%
- 75%
- 100%

**Distance and Visibility:**

**Distance from Nearest Sunscreen Dispenser:** __________ meters

**Notes on Dispenser location:**

Visible:

- Yes
- No

Accessible:

- Yes
- No

**Instruction:**

Tally applicable categories for each observed individual.

**Appendix J**

**Sun-Protection Behaviour Dispenser Observation Tool (Observation tool 2)**

**Location of Dispenser:** _____________________________________________________________
**Date:** _______________
**Time Interval:** _______________

**Observation Number: ________**

**Weather and Environmental Conditions:**

- **UV Index:** ___________
- **Temperature:** ________°C
- **Cloud Coverage:**
  - 0%
  - 25%
  - 50%
  - 75%
  - 100%

**Notes on Dispenser location:**

Visible:

- Yes
- No

Accessible:

- Yes
- No

Ground Type

- Paved location
- Grass location

Location Type

- Beach location
- Park location

**Instructions:**

- Mark Y= Yes, N= No, NA= Unable to observe for each sun safety behaviour observed individual.

| **Entry** | **User Count** | **Group ID** | **Age Group (Child/Teens/Adult)** | **Hender** | **Sunglasses** | **No Hat** | **Hat - Visor** | **Hat - Cap** | **Hat - Broad Brim** | **Shirt – Sleeveless to ¼** | **Shirt – ½ Length** | **Shirt - ¾ to full** | **Leg - Bikini/Shorts** | **Leg - Mid-thigh/Knee** | **Leg - Full Cover** | **Sunscreen - Arms** | **Sunscreen - Face** | **Sunscreen – Chest** | **Sunscreen-**  **Back** | **Sunscreen – Thighs and lower legs** | **Sunscreen Amount (S/M/L)** | **Shoes - Closed-Toe** |
| --- | --- | --- | --- | --- | --- | --- | --- | --- | --- | --- | --- | --- | --- | --- | --- | --- | --- | --- | --- | --- | --- | --- |
| 1 | 1 | G1 | Adult | M/F |  |  |  |  |  |  |  |  |  |  |  |  |  |  |  |  | S/M/L |  |
| 2 | 2 | G1 | Teens | M/F |  |  |  |  |  |  |  |  |  |  |  |  |  |  |  |  | S/M/L |  |
| 3 | 3 | G1 | Child | M/F |  |  |  |  |  |  |  |  |  |  |  |  |  |  |  |  | S/M/L |  |
| 4 | 4 | G1 | Teens | M/F |  |  |  |  |  |  |  |  |  |  |  |  |  |  |  |  | S/M/L |  |
| 5 | 5 | G2 | Adult | M/F |  |  |  |  |  |  |  |  |  |  |  |  |  |  |  |  | S/M/L |  |
| 6 | ... | ... | ... | M/F |  |  |  |  |  |  |  |  |  |  |  |  |  |  |  |  | S/M/L |  |
| 7 | 100 | G10 | Senior | M/F |  |  |  |  |  |  |  |  |  |  |  |  |  |  |  |  | S/M/L |  |
| 8 |  |  |  | M/F |  |  |  |  |  |  |  |  |  |  |  |  |  |  |  |  | S/M/L |  |
| 9 |  |  |  | M/F |  |  |  |  |  |  |  |  |  |  |  |  |  |  |  |  | S/M/L |  |
| 10 |  |  |  | M/F |  |  |  |  |  |  |  |  |  |  |  |  |  |  |  |  | S/M/L |  |
| 11 |  |  |  | M/F |  |  |  |  |  |  |  |  |  |  |  |  |  |  |  |  | S/M/L |  |

**Appendix K**

**Table 11.** Associations between sun-protective behaviours and vicinity to sunscreen dispensers among individuals observed in public outdoor settings in Toronto, July to September 2024, estimated using Fisher’s exact test.

| Sun Safety Behaviour | OR | CI |
| --- | --- | --- |
| Sunglasses | 0.99 | (0.76, 1.28) |
| Hats | 0.86 | (0.67, 1.10) |
| Long Shirt | 0.95 | (0.70, 1.28) |
| Long Pants | 1.16 | (0.90, 1.50) |
| Portable shade | 0.93 | (0.69, 1.25) |
| Natural shade | 1.45 | (1.13, 1.83) |

Odds ratios (OR) and 95% confidence intervals (CI) were estimated using Fisher’s exact test.

Values greater than 1 indicate higher odds of the behaviour among individuals observed near sunscreen dispensers compared with those observed farther from dispensers.

**Appendix L**

**Table 12.** Associations between sun-protective behaviours and demographic and temporal factors among sunscreen dispenser users observed in public outdoor settings in Toronto, July to September 2024, estimated using Fisher’s exact test.

| Behaviour^a^ | OR | CI |
| --- | --- | --- |
| Sunglasses |  |  |
| Gender | 3.545 | (1.626, 7.732) |
| Age Group | N/A | N/A |
| Weekend | 2.604 | (1.196, 5.669) |
| Time period | 0.618 | (0.263, 1.451) |
| Cap |  |  |
| Gender | 1.023 | (0.467, 2.24) |
| Age Group | N/A | N/A |
| Weekend | 0.583 | (0.285, 1.193) |
| Time period | 1.078 | (0.491, 2.368) |
| Sleeveless to ¼ |  |  |
| Gender | 0.730 | (0.19, 2.803) |
| Age_Group | N/A | N/A |
| Weekend | 0.462 | (0.143, 1.492) |
| Time period | 3.464 | (1.084, 11.067) |
| ½ length shirt |  |  |
| Gender | 1.435 | (0.687, 2.997) |
| Age_Group | N/A | N/A |
| Weekend | 1.615 | (0.824, 3.166) |
| Time period | 0.656 | (0.31, 1.385) |
| ¾ to full length shirt |  |  |
| Gender | 1.00 | (0.464, 2.157) |
| Age Group | N/A | N/A |
| Weekend | 1.243 | (0.615, 2.511) |
| Time period | 1.433 | (0.669, 3.07) |
| Bikini Shorts |  |  |
| Gender | 1.128 | (0.54, 2.356) |
| Age Group | N/A | N/A |
| Weekend | 0.974 | (0.499, 1.899) |
| Time period | 1.409 | (0.667, 2.977) |
| Mid-thigh to Knee length pants |  |  |
| Gender | 1.741 | (0.464, 6.531) |
| Age_Group | N/A | N/A |
| Weekend | 0.781 | (0.216, 2.828) |
| Time period | 0.269 | (0.033, 2.197) |
| Full Cover pants |  |  |
| Gender | 0.744 | (0.347, 1.592) |
| Age Group | N/A | N/A |
| Weekend | 1.101 | (0.557, 2.178) |
| Time period | 0.915 | (0.429, 1.951) |
| Sunscreen Arms |  |  |
| Gender | N/A | N/A |
| Age Group | N/A | N/A |
| Weekend | N/A | N/A |
| Time period | N/A | N/A |
| Sunscreen Face |  |  |
| Gender | 1.303 | (0.483, 3.514) |
| Age Group | N/A | N/A |
| Weekend | 0.543 | (0.213, 1.388) |
| Time period | 0.565 | (0.177, 1.798) |
| Sunscreen on Thighs and Lower Legs |  |  |
| Gender | 2.345 | (0.736, 7.472) |
| Age Group | N/A | N/A |
| Weekend | 1.303 | (0.404, 4.201) |
| Time of day | 1.710 | (0.523, 5.588) |

**^a^** Behaviours are from the observational tool 2 that observed sun protective behaviours of sunscreen dispenser users.

Odds ratios (OR) and 95% confidence intervals (CI) were estimated using Fisher’s exact test.

N/A = Not possible to estimate due to lack of variability in the behaviour

**Appendix M**

**Table 13.** Source citation chart of each variable used for data collection

| Data Point | Description | Source Citation |
| --- | --- | --- |
| User Count | Count of individuals observed during each entry | Dixon et al., 2008; Dexter et al., 2009 |
| Group ID | Identification number for groups observed | Dixon et al., 2008; Dexter et al., 2009 |
| Demographic Group (Child/Teens/Adult) | Estimated age group of the observed individuals | Milne et al., 1999, Dobbinson et al.2014, McKenzie et al. 2000 |
| Gender (M/F) | Gender of the observed individuals | Wood et al., 2017, Dobbinson et al., 2014 |
| Group Composition (Individual/Group) | Observing whether individuals are alone or in groups | Dixon et al., 2008; Dexter et al., 2009 |
| Sunglasses | Use of sunglasses as a sun protection measure | ORiordan et al. 2009 |
| Hat - No Hat | Observation of hat usage | Maddock et al., 2007, Buller et al. 2017, Wood et al., 2017, Lanerlund et al., 2006 |
| Hat - Visor | Specific type of hat observed | Maddock et al., 2007, Buller et al. 2017, Wood et al., 2017, Lanerlund et al., 2006 |
| Hat - Cap | Another specific type of hat observed | Maddock et al., 2007, Buller et al. 2017, Wood et al., 2017, Lanerlund et al., 2006 |
| Hat - Broad Brim | Broad-brimmed hats observed | Maddock et al., 2007, Buller et al. 2017, Wood et al., 2017, Lanerlund et al., 2006 |
| Shirt - Sleeveless to ¼ Length | Type of shirt worn sleeveless to ¼ length | ORiordan et al. 2009, Buller et al. 2017, Nikles & Harrison, 2013 |
| Shirt - ½ | Observation of ½ length sleeved shirts | ORiordan et al. 2009, Buller et al. 2017, Nikles & Harrison, 2013 |
| Shirt - ¾ Length to full | Observation of ¾ length and longer | ORiordan et al. 2009, Buller et al. 2017, Nikles & Harrison, 2013 |
| Leg - Bikini/Shorts | Leg cover type indicating sun exposure | Maddock et al., 2007, Buller et al. 2017, Wood et al., 2017, Lanerlund et al., 2006 |
| Leg - Mid-thigh/Knee | Mid-thigh to knee coverage observed | Maddock et al., 2007, Buller et al. 2017, Wood et al., 2017, Lanerlund et al., 2006 |
| Leg - Full Cover | Full leg coverage observed | Dixon et al., 2008, Buller et al. 2017, Wood et al., 2017, Lanerlund et al., 2006 |
| Sunscreen - Arms | Sunscreen application on arms | Wood et al., 2017 |
| Sunscreen - Face | Sunscreen application on face observed | Wood et al., 2017 |
| Sunscreen - Chest | Sunscreen application on chest | Wood et al., 2017 |
| Sunscreen - Back | Sunscreen application on back | Wood et al., 2017 |
| Sunscreen - thigh/lower legs | Sunscreen application on thigh/lower legs | Wood et al., 2017 |
| Shoes - Closed-Toe shoes | Observation of closed-toe shoes | Dixon et al., 2008, Buller et al. 2017 |
| Shoes - Open-Toed shoes | Observation of open-toed shoes | Dixon et al., 2008, Buller et al. 2017, |
| UV index | The UV index at the time of observation | Dixon et al., 2008, Buller et al. 2017, Dudley et al., 2017 |
| Temperature | The temperature at the time of observation | Dixon et al., 2008, Buller et al. 2017, Dudley et al., 2017 |
| Visibility | A sunscreen dispenser was considered "visible" if it was unobstructed and easily seen from points like benches or walking paths, without requiring to turn around or shift positions. | Cure & Van Enk, 2015 |
| Accessibility | A sunscreen dispenser is 'accessible' if can be approached and use it without physical barriers (e.g., fencing, landscaping, or seating obstructions). | Cure & Van Enk, 2015; Keurentjes et al., 2022 |
| Ground Type | The type of ground the dispenser is mounted based paved (concrete, asphalt) or grass (turf or natural grass areas) | Vanos et al., 2017; Ferro-Sánchez et al., 2023 |
| Location Type | The type of location the dispenser is mounted. Location was classified as 'beach' if the area provided allows direct access to water for recreational use, and as 'park' if not and was primarily used for land-based activities. | Lanerlund et al., 2006 |
| Weekday | Dispenser observation assessed on weekday | Heerfordth et al., 2018 |
| Weekend | Dispenser observation assessed on weekend | Heerfordth et al., 2018 |
| Zone locations | Whether 250m close to dispenser (close vicinity) or farther away (Far vicinity) | Hunter et al., 2018, Farag et al., 2024 |
| Visibility of dispenser | Whether the nearest dispenser is clearly visible, partially or obscured | Cure & Van Enk, 2015 |

**Appendix N**

**Table 14.** Distribution of sun-protective behaviours among sunscreen dispenser users by gender observed in public outdoor settings in Toronto, July to September 2024.

| Gender | Sunglasses Count | No Hat Count | Cap Count | Broad Brim Count | Sleeveless to ¼ shirt Count | Half Length shirt Count | Three Quarter Length shirt to Full Count | Bikini Shorts Count | Mid-thigh Knee pants Count | Full Cover pants Count | Sunscreen Arms Count | Sunscreen Face Count | Sunscreen Back Count | Sunscreen Thighs Lower Legs Count | Closed Toed Shoes Count |
| --- | --- | --- | --- | --- | --- | --- | --- | --- | --- | --- | --- | --- | --- | --- | --- |
| Women | 22 | 62 | 32 | 1 | 10 | 46 | 35 | 52 | 6 | 42 | 97 | 14 | 1 | 7 | 71 |
| Men | 20 | 25 | 13 | 0 | 3 | 22 | 14 | 22 | 4 | 14 | 40 | 7 | 0 | 6 | 32 |

**Table 15.** Distribution of sun-protective behaviours among sunscreen dispenser users by age group observed in public outdoor settings in Toronto, July to September 2024.

| Age Group | Sunglasses Count | No Hat Count | Cap Count | Broad Brim Count | Sleeveless to ¼ shirt Count | Half Length shirt Count | Three Quarter Length shirt to Full Count | Bikini Shorts Count | Mid-thigh Knee pants Count | Full Cover pants Count | Sunscreen Arms Count | Sunscreen Face Count | Sunscreen Back Count | Sunscreen Thighs Lower Legs Count | Closed Toed Shoes Count |
| --- | --- | --- | --- | --- | --- | --- | --- | --- | --- | --- | --- | --- | --- | --- | --- |
| Adult | 41 | 71 | 29 | 1 | 9 | 54 | 40 | 55 | 10 | 41 | 103 | 14 | 1 | 10 | 75 |
| Child | 0 | 5 | 3 | 0 | 2 | 3 | 3 | 3 | 0 | 5 | 8 | 6 | 0 | 0 | 4 |
| Teen | 1 | 11 | 13 | 0 | 2 | 11 | 6 | 16 | 0 | 10 | 26 | 1 | 0 | 3 | 24 |

**Appendix O**

**Table 16.** Adjusted incidence rate ratios and 95% confidence intervals examining the interaction between vicinity to sunscreen dispensers and age group on sun-protective behaviours among individuals observed in public outdoor settings in Toronto, July to September 2024.

| Variables | Sun Protective Behaviours^a^ | | | | | |
| --- | --- | --- | --- | --- | --- | --- |
|  | Sunglasses | Hats | Long Shirt | Long Pants | Portable Shade | Natural Shade |
| Vicinity |  |  |  |  |  |  |
| Close | 0.85 (0.57, 1.26) | 0.87 (0.55, 1.38) | 1.00 (0.52, 1.93) | 1.17 (0.76, 1.81) | 1.09 (0.29, 4.01) | 1.09 (0.57, 2.05) |
| Demographic Group |  |  |  |  |  |  |
| Children | 0.02 (0.01, 0.06) | 0.14 (0.08, 0.26) | 0.09 (0.04, 0.24) | 0.13 (0.07, 0.24) | 0.24 (0.06, 0.96) | 0.30 (0.15, 0.58) |
| Teen | 0.13 (0.08, 0.23) | 0.11 (0.06, 0.21) | 0.39 (0.19, 0.78) | 0.39 (0.24, 0.63) | 0.17 (0.04, 0.71) | 0.35 (0.18, 0.68) |
| Vicinity × Demographic (interaction term) |  |  |  |  |  |  |
| Vicinity Close: Children | 1.90 (0.42, 8.61) | 1.44 (0.65, 3.22) | 2.15 (0.62, 7.39) | 0.85 (0.35, 2.09) | 0.35 (0.05, 2.64) | 1.02 (0.39, 2.62) |
| Vicinity Close: Teen | 0.88 (0.39, 2.00) | 0.86 (0.35, 2.14) | 0.40 (0.13, 1.19) | 0.46 (0.22, 0.95) | 1.28 (0.18, 9.13) | 0.64 (0.24, 1.66) |
| UV Index | 1.15 (1.01, 1.31) | 1.21 (1.06, 1.37) | 1.17 (0.98, 1.40) | 0.94 (0.83, 1.06) | 1.48 (1.09, 2.01) | 1.16 (1.00, 1.36) |
| Cloud Coverage | 0.99 (0.98, 1.00) | 0.99 (0.98, 1.00) | 1.00 (0.98, 1.00) | 1.00 (0.99, 1.00) | 1.58 (1.10, 2.27) | 1.00  (0.99, 1.01) |

**^a^** Behaviours are from the observational tool 1 that observed sun protective behaviours in the far and close vicinity to dispensers.

Behaviours were measured using Observation Tool 1 among individuals observed in areas near and far from sunscreen dispensers. Interaction terms (Vicinity x Demographic) represent the combined effect of vicinity and age group on each behaviour, compared with the reference group (adults observed farther from dispensers).

**Appendix P**

**Mean Users per Dispenser by Ground Type**


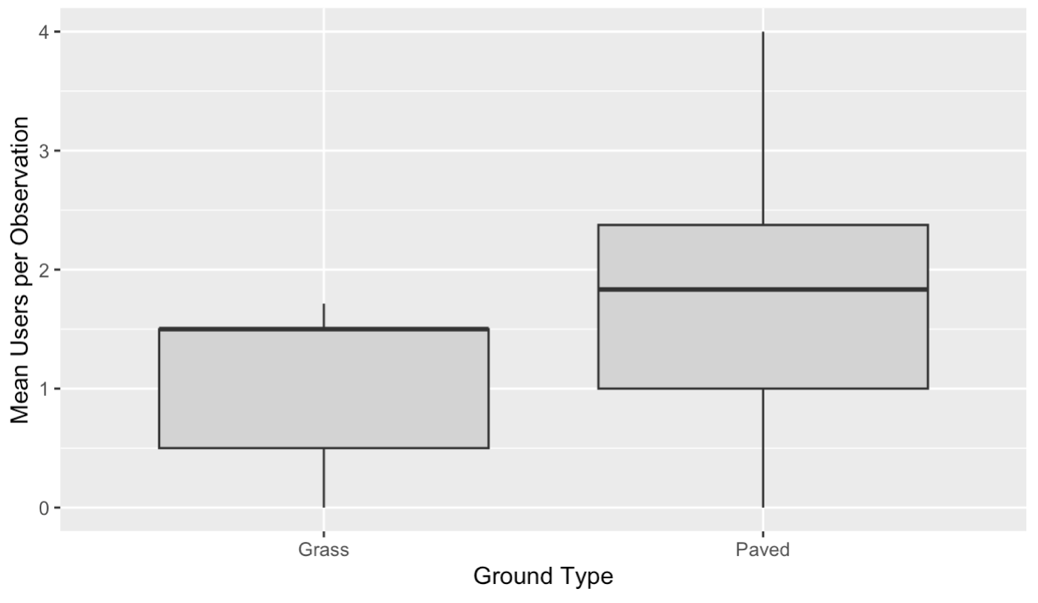


**Figure 4.** Distribution of mean users per sunscreen dispenser by ground type (grass and paved) among sites observed in public outdoor settings in Toronto, July to September 2024.

**Mean Users per Dispenser by Visibility Type**


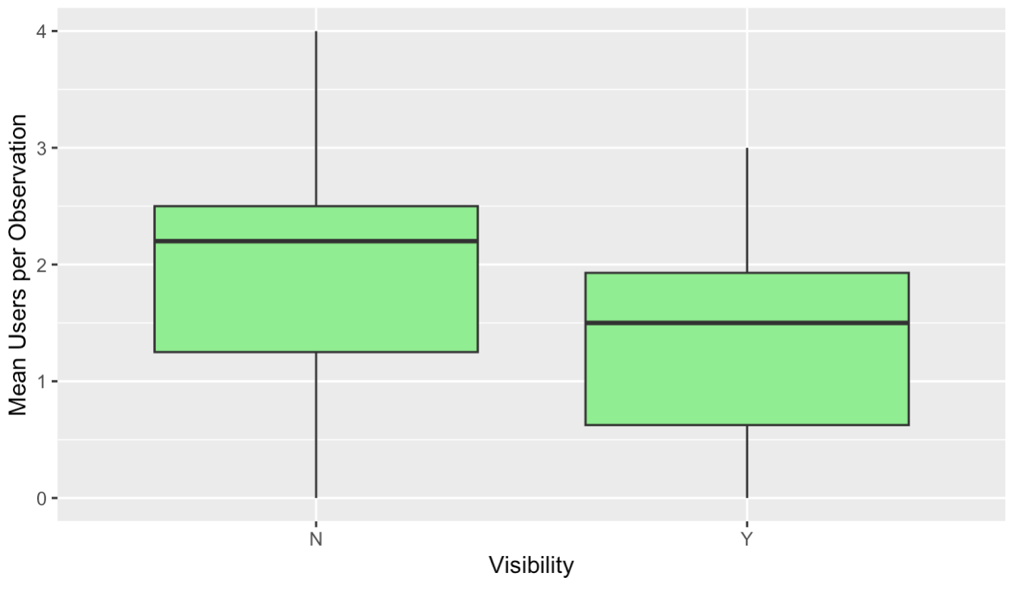


**Figure 5.** Distribution of mean users per sunscreen dispenser by visibility (visible and not visible) among sites observed in public outdoor settings in Toronto, July to September 2024.


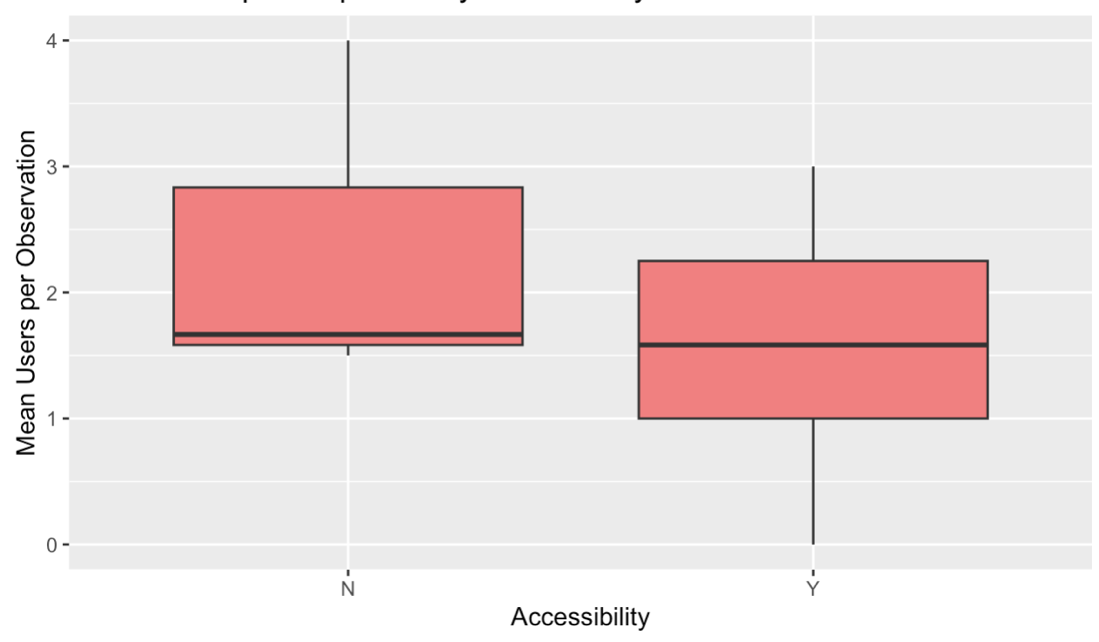


**Mean Users per Dispenser by Accessibility Type**

**Figure 6.** Distribution of mean users per sunscreen dispenser by accessibility (accessible and not accessible) among sites observed in public outdoor settings in Toronto, July to September 2024.

**Mean Users per Dispenser by Location Type**


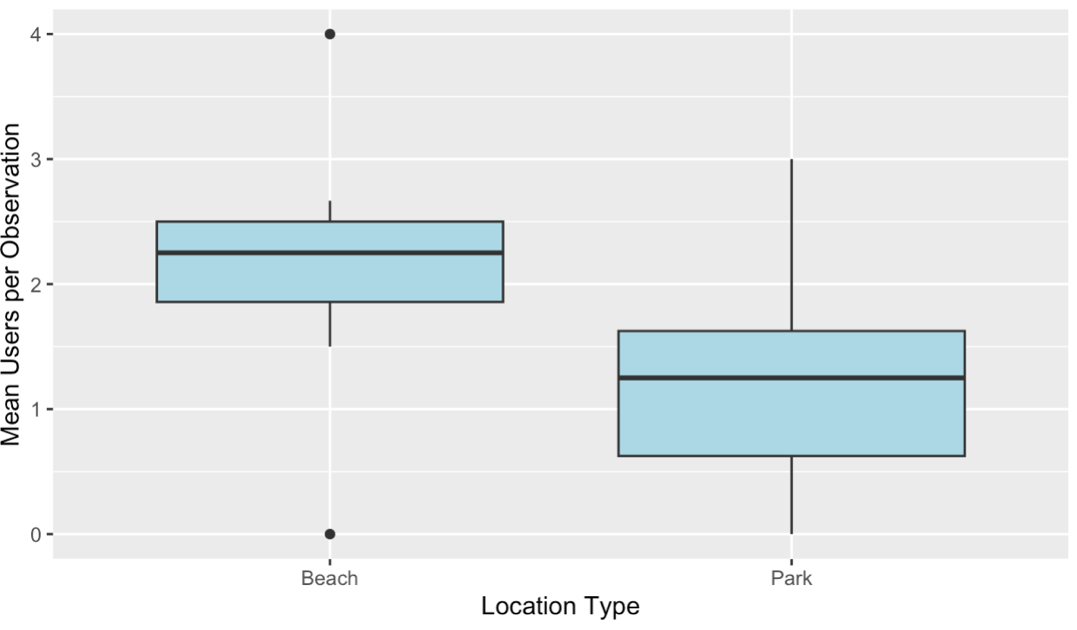


**Figure 7.** Distribution of mean users per sunscreen dispenser by location type (park and beach) among sites observed in public outdoor settings in Toronto, July to September 2024.
